# Supplementary material for: Content-rich biological network constructed by mining PubMed abstracts
Source: BMC Bioinformatics. 2004 Oct 8;5:147. doi: 10.1186/1471-2105-5-147 (PMC528731; doi:10.1186/1471-2105-5-147)
Supplement: Additional File 2 — The original results of the above study (non-essential files are deleted to keep the file size under the limit set by BMC bioinformatics). [file 1471-2105-5-147-S2.bz2 › chilibotAdditionalFile2/dip05/129ID7644510E474/html/CDC25A_YWHAE.html]

 


 **CDC25A** and **YWHAE** 
  
Found 1 abstracts in PubMed, retrieved 1.  
 

 What does Google say? 
 PDF only 
| .edu only 

---

- Proc Natl Acad Sci U S A, 1995   **14 3 3 proteins associate with cdc25 phosphatases.** .
  The cdc25 phosphatases play key roles in cell cycle progression by activating cyclin dependent kinases.
  Two members of the 14 3 3 protein family have been isolated in a yeast two hybrid screen designed to identify proteins that interact with the human **cdc25A** and cdc25B phosphatases.
  Genes encoding the human homolog of the 14 3 3 epsilon **[YWHAE]** protein and the previously described 14 3 3 beta protein have been isolated in this screening.
  14 3 3 proteins constitute a family of well conserved eukaryotic proteins that were originally isolated in mammalian brain preparations.
  possess diverse biochemical activities related to signal transduction.
  We present evidence that indicates that cdc25 and 14 3 3 proteins physically interact both in vitro and in vivo.
  14 3 3 protein does not affect the phosphatase activity of **cdc25A**.
  Raf 1, which is known to bind 14 3 3 proteins, has recently been shown to associate with **cdc25A** and to stimulate its phosphatase activity.
  14 3 3 protein has no effect on the **cdc25A** kinase activity of Raf 1.
  Instead, 14 3 3 may facilitate the association of cdc25 with Raf 1 in vivo, participating in the linkage between mitogenic signaling and the cell cycle machinery.
